# Adsorption, Equilibrium Isotherm, and Thermodynamic Studies towards the Removal of Reactive Orange 16 Dye Using Cu(I)-Polyaninile Composite

**DOI:** 10.3390/polym13203490

**Published:** 2021-10-11

**Authors:** Prasanna Kumar Obulapuram, Tanvir Arfin, Faruq Mohammad, Sachin K. Khiste, Murthy Chavali, Aisha N. Albalawi, Hamad A. Al-Lohedan

**Affiliations:** 1 Wits Advanced Drug Delivery Platform Research Unit, Department of Pharmacy and Pharmacology, School of Therapeutic Sciences, Faculty of Health Sciences, University of the Witwatersrand, Johannesburg, 7 York Road, Johannesburg 2193, South Africa; obulapuram1727@gmail.com; 2 OPK Tech Solutions (PTY) Ltd., Pharmaceutical and Advanced Drug Delivery Research, 69 Hamlin Street, Highlands North, Johannesburg 2192, South Africa; 3 Hyderabad Zonal Centre, CSIR-National Environmental Engineering Research Institute (CSIR-NEERI), IICT Campus, Tarnaka, Hyderabad 500007, India; t_arfin@neeri.res.in; 4 Department of Chemistry, College of Science, King Saud University, Riyadh 11451, Saudi Arabia; hlohedan@ksu.edu.sa; 5 Department of Medicine, Harvard Medical School, Boston, MA 02115, USA; skhiste@bwh.harvard.edu; 6 Office of the Dean (Research) & Division of Chemistry, Department of Science, Faculty of Science & Technology, Alliance University, Chandapura-Anekal Main Road, Bengaluru 562106, India; Siva.Chavali@alliance.edu.in; 7 NTRC-MCETRC and 109 Composite Technologies Pvt. Ltd., Guntur District, Guntur 522201, India; 8 Department of Chemistry, University College of Haql, University of Tabuk, Tabuk 71491, Saudi Arabia; an.albalawi@ut.edu.sa

**Keywords:** wastewater treatment, adsorption isotherms, reactive orange 16, Cu(I)-polyaniline, thermodynamics

## Abstract

To overcome some of the limitations of activated carbon like efficiency, cost-effectiveness, and reusability, the present work deals with Cu(I)-based polyaniline (PANI) composite for the removal of reactive orange 16 (RO16) dye. Following the synthesis and characterization of formed Cu(I)-PANI composite, the batch experiments performed for the removal of RO16 dye indicated that the composite has the capacity to reduce the coloring from RO16. The experiments were conducted for the study of effects against changes in pH, time, and dose at room temperature, where we observed for a pH impact on the dye adsorption capacity in the range of 2–12. Among all, the optimal RO16 removal was found to be 94.77% at a pH of 4 and in addition, the adsorption kinetics confirmed to be pseudo-second-order with more suitability towards the Langmuir isotherm, where it is presumed to be the formation of a monolayer of dye molecule at the homogeneous absorbent surface. The calculated maximum capacity, q_m_, determined from the Langmuir model was 392.156 mg/g. Further application of isotherms to attain thermodynamic parameters, a slight positive value of Δ*S*° for RO16 adsorption was observed, meaning that there is an increased randomness in the irregular pattern at the specific Cu(I)-PANI interface for an adsorption process. This mechanism plays an essential role in maintaining the effects of water pollution; and, based on the analysis therefore, it is prominent that the Cu(I)-PANI composite can be employed as a promising and economical adsorbent for the treatment of RO16 and other dye molecules from the sewage in wastewater.

## 1. Introduction

In recent years, the increased demand to obtain pure drinking water with only limited resources has garnered interest in the development of new technologies for water purification and in upgrading the traditional water management processes. The usage after the recycling of wastewater generated from the industry indicates the importance of developing and enhancing the water management schemes that cover critical aspects. The significant parameters of contamination for elaborating wastewater are loss of transparency and the attaining of color [1]. The leading cause of water poisoning is the release of sewage water into the rivers and other water streams that contain severely high levels of dyes from fabric, paint, paper, wood, and metal industries, far surpassing the permissible amounts [2]. In particular, the textile industry makes use of dye cellulose fibers as an alternative to the reactive dyes and they are characterized through the N=N bond, where the coloring of such dyes is because of the azo bond linked to the chromophores. The functional group and chromophore are responsible for the binding of dyestuff with that of cellulose fiber. The reaction occurs due to the formation of a covalent bond which is highly resistant to harsh environmental conditions as compared to the other physicochemical bonds between the dyes and cellulose fibers. The hydrolyzed dyes (%) have a hydroxyl ion that does not react with the cellulose fiber and so, approximately 10–50% of the initial dye load is available in the dye and in that way they have the ability to form highly colored effluent [3]. The main component in the structure is aromatic rings and the dye does not thoroughly biodegrade. It is also observed that the toxic and carcinogenic effects of respective dyes have severe impacts on aquatic life [4]. The main problem with the reactive dyes is that they cannot be removed quickly by means of the typical wastewater treatment processes; this is due to their fundamental features: namely, high pH and salt concentration, resistance to light, stability etc. Therefore, it is highly important to remove the azo dyes from the wastewater effluent before they are released into the reservoirs, rivers, and other water streams. 

The present studies are concentrated on the growth of efficient processes to remove organic contaminants from wastewater and textile industry effluents [5]. The conventional approaches, namely, coagulation, precipitation, and flocculation that are employed for the treatment of wastewater on site, are not capable of removing most of the dyes certainly from the dilute solution [6]. In that view, photo-oxidation is a recognized approach and functions by means of treating the dye-containing effluents; as such, the respective process is costly and unsuitable for handling large flows [7]. Also, the biological degradation is considered as an adequate alternative for the reactive dye decolorization [8]. However, purification by adsorption is a commonly used approach for the decontamination of effluents generated from textile industries. It was observed that the adsorption was superior in comparison to various other techniques practiced for the purification of industrial and sewage water. The advantages associated with this technique include cost, specificity, ease, simplicity of operation, fewer factors to consider, and the capability to treat dye effluent even at high concentrations [9]. In this adsorption technique, many different solid adsorbents with varying sizes, shape, surface charges, porosity, etc. are being employed for the decontamination of water. Some of the commercial systems employ activated carbons, the organic resin in the form of adsorbents for the removal of dye in the effluent due to the capability of efficient adsorption [10]. The activated carbon is a vigorously applied adsorbent for removing color and treatment of effluents generated from textiles due to the well-organized pore structure, and the reflective surface causes high adsorption capacity. Still, the activated carbon has specific limitations such as high price, which led to the search of a cheap, efficient, and readily available adsorbent material [11]. To overcome the limitations of activated carbon, various base materials are being developed to fulfil the requirements of economical and efficient adsorbents originating from non-conventional agricultural and industrial practices. 

Polyaniline (PANI) attracted interest because of features like ease of synthesis, low cost, electrical conductivity, and environmental constancy. It exhibited excellent adsorption ability because of the large number of imine and amine functional groups in the polymer chain, which enables it to be employed as an adsorbent during the removal of dyes from wastewater solution [12]. Ai et al. [13] used a homogeneous PANI microsphere in the form of an adsorbent to remove methyl orange (MO) dye and from the study, the adsorption capacity of PANI microsphere was observed to be 154.56 mg/g. Other similar studies were also carried out towards the adsorption of methylene blue (MB) dye molecules onto PANI nanotubes where the adsorption capacity of 4.8 mg/g was noted. [14]. Gopal et al. [15] also used PANI/AC for removing direct red 23 (DR23) from the solution and the adsorption capacity was found to be 109.89 mg/g at pH 3.

Similarly, for adsorption-related applications, the structure of copper shows some unusual behavior that supports for a favorable material activity such as size, shape, and surface activity [16,17]. Therefore, taking advantage of the fundamental properties of both Cu and PANI, the present study is aimed to test the adsorption efficacy of Cu(I)-PANI composite towards the removal of reactive orange 16 (RO16) dye. For the synthesis of Cu(I)-PANI supra-molecular composite, the in-situ polymerization and composite formation (IPCF) technique was employed [18], and this technique employs copper sulfate in the form of an oxidizing agent to the polymeric aniline. For the polymerization, the single step is co-related to the discharge of electrons resulting in the reduction of Cu(II) ions forming Cu(I) ions. The Cu(I) ions are the primary center to bind the chain of nitrogen to PANI. Therefore, the significant target is introducing Cu(I)-PANI composite as an excellent adsorbent in the textile dye, RO16. The various parameters were employed to investigate the physicochemical characteristics (such as powdered XRD, XPS, UV-Vis, TGA, DSC, and BET), followed by the analysis towards adsorption isotherms, reaction kinetics, and thermodynamic studies. For the Cu(I)-PANI composite testing, the dosage concentration, contact time, adsorption isotherm, adsorption kinetics, thermodynamic parameters, and recycling studies towards the removal of RO16 dye are being investigated.

## 2. Materials and Methods

### 2.1. Materials 

The chemicals used to synthesize Cu(I)-PANI, including aniline, methanol, and copper sulfate, were purchased from Sigma-Aldrich (Mumbai, India), as they are similar to Reactive orange 16 (RO16) dye. All the reagents used were of highest standards and are analytical grade; all the solutions were made using deionized water solvent.

### 2.2. Instrumentation

For the characterization of Cu(I)-PANI composite, the powder x-ray diffraction (XRD) was carried out on an XRD 6000 instrument (Shimadzu, Kyoto, Japan), and the UV-Vis spectroscopy analysis was studied using a SPECORD PLUS double beam spectrophotometer (Analytikjena, Jena, Germany). Also, the elemental analysis and their oxidation states was investigated using the X-ray photoelectron spectroscopy (XPS), and for that, AXIS 165 high performance multi-technique surface analyzer with a mean radius hemisphere of 165 mm and an eight channeltron detection system was used. The BET surface area was observed by mercury intrusion porosimetry (Model AutoPore IV 9500 V, Micromeritics, Norcross, GA, USA). The thermogravimetric analysis (TGA) and differential scanning calorimetry (DSC) were studied to investigate the thermal stability of the composite and for that, a Perkin-Elmer instrument was used.

### 2.3. Synthesis of Cu(I)-PANI Composite

The synthesis procedure used for the formation of Cu(I)-PANI composite was the slight modification of an earlier reported method elsewhere [19]. Briefly, 0.01 M of aniline solution was diluted in 25 mL of CH_3_OH in a conical flask followed by the addition of 0.01 M of CuSO_4_·5H_2_O solution in distilled water, where the molar concentration ratio was maintained to 1:1 while stirring continuously for 24 h. First, the mixture at the time of addition was green in color and by the end of 24 h of stirring, the formation of parrot-green precipitation at the bottom of the flask can be noted. The complete reaction process was carried out at room temperature and at the normal atmospheric pressure. The material was filtered out and repeatedly washed using deionized water, and then dried at 50 °C for 24 h in a vacuum.

### 2.4. Preparation of Dye Solution

The RO16 dye was employed to test the adsorption capacity of the as-synthesized Cu(I)-PANI composite. For the testing, 0.5 g of dye powder was dissolved in 1000 mL distilled water to prepare a stock solution of 500 mg/L concentration. The diverse concentration of dye solution proceeded from the suitable dilution of stock solution with the distilled water. 

### 2.5. Calibration Curve

The dilution of stock solution was used to prepare five samples of RO16 with different concentrations (20, 40, 60, 80, and 100 mg/L). The maximum RO16 wavelength and the absorbance value of samples were taken into account by the UV-Vis spectra, and a calibration curve was established. The absorbance value received from the calibration curve of absorbance was 494 nm, and it was made at the maximum RO16 wavelength. According to IUPAC (International union of pure and applied chemistry) guidelines, the RO16 detection limit was 0.12 mg/L when using the spectrophotometric method [20].

### 2.6. Reusability

The cycles of adsorption-sorption were carried out repeatedly for five times to investigate the reusability of the synthesized composite and its adsorption capacity. The adsorbent was even reacted with 10 mL ethanol at room temperature.

## 3. Results

### 3.1. Physicochemical Studies

**Figure 1:**
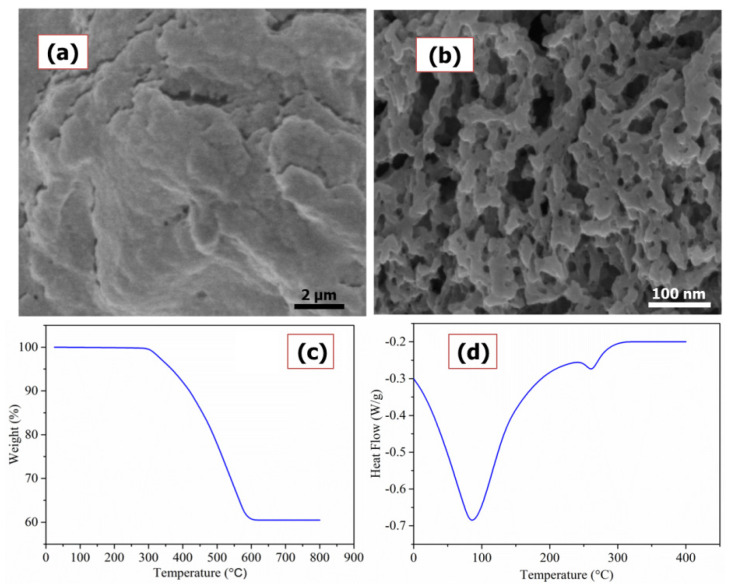
SEM image of Cu(I)-PANI composite at two different scales of (**a**) 2 µm and (**b**) 100 nm; (**c**,**d**) corresponds to the TGA and DSC analysis of the same Cu(I)-PANI composite (respectively).

Figure 1a,b shows the comparison of the SEM images of Cu(I)-PANI composite at two different magnifications (scales of 2 µm and 100 nm) and from the figure, it can be noticed that the PANI surface is soft and porous. 

These surface-porous sites may serve as the stable houses for the localization of adsorbent molecules when the Cu(I)-PANI composite is employed for adsorption-related applications. Further, the TGA was used for testing the thermal stability of Cu(I)-PANI composite where the material was kept to rest at 30 °C for 1 min, and, after that, it was heated between 30 and 600 °C at 10 °C/min (Figure 1c). As shown in the figure, there is a loss of mass to the Cu(I)-PANI composite when heated in an atmosphere of nitrogen; we observed a weight loss to the Cu(I)-PANI composite at first below 298 °C, referring to the removal of physisorbed water and dopant ions. The subsequent loss observed in the range of 300 to 590°C corresponds to the degradation of the polymer backbone. This weight loss can be assigned to the expulsion of water from the structure of the polymer, but at a higher temperature, the degradation of the polymer chain occurs because of the dopant. On dopant removal, the decomposition of polymer skeleton takes place at an extreme temperature [21]. The weight loss of composite above 590 °C temperature was only 1.28%, meaning that the Cu(I) addition is restricting the degradation of PANI. Based on the analysis, the TGA shows a low thermal stability of Cu(I)-PANI composite because of the inculcation of Cu(I) particles in the form of dopant at the N_2_ sites of PANI [22]. 

DSC can be employed to study the glass transition and melting behavior of any sample. Figure 1d shows the DSC analysis of Cu(I)-PANI composite. From the graph, it is observed that the PANI shows only one endothermic peak at 85 °C, which is mainly due to the vaporization of water added to the glass transition behavior [23]. The importance of the glass transition temperature on the assimilation of the inorganic phase in the polymer matrix by in-situ polymerization is associated with excellent interfacial interaction between the particles and polymer, causing a restriction on the mobility of the PANI chain. Also, the graph shows a weak endothermic dip at 260 °C temperature, which could be from the melting of the PANI chain. It can also be observed that the onset of thermal degradation at a low temperature along with doping exhibits that the metal cations that are adsorbed on the surface of the molecule may be affected very quickly. This section may be divided by subheadings. It should provide a concise and precise description of the experimental results, their interpretation, as well as the experimental conclusions that can be drawn.

**Figure 2:**
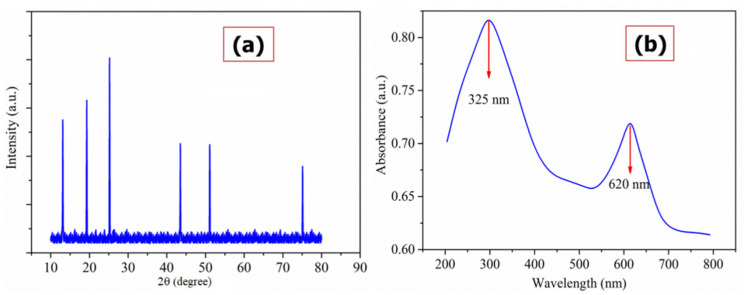
(**a**) Powdered XRD patterns and UV-Vis (**b**) spectroscopic analysis of the Cu(I)-PANI composite.

The XRD is non-destructive, and it is an excellent technique for identifying the crystallinity of solid materials; the powdered XRD of Cu(I)-PANI composite is shown in Figure 2a, where the diffraction patterns of PANI are observed at 2θ of 13.08°, 19.3°, and 25.2° [24]. Similarly, the Cu’s diffraction patterns are observed around 43.3°, 51.1°, and 75.1° corresponding to the plans of (111), (200), and (311) respectively, indicating for the presence of particles in the face-centered cubic lattice crystals [25]. It can also be noticed for the non-observation of CuSO_4_ peaks in the XRD pattern, meaning that the Cu particles are reduced during the polymerization process.

Figure 2b shows the UV-Vis spectroscopy of Cu(I)-PANI composite, as this is a very essential technique for the characterization of interfacial interactions between CuSO_4_ and PANI. From the spectrum, two different absorption peaks can be observed in the wavelength range of 320–330 nm and 600–660 nm which can be linked to the corresponding transitions of π-π* and n-π* (respectively) of benzenoid and quinoid rings [26]. In particular, the peak observed at 325 nm refers to the π-π* transition of benzenoid, and 620 nm for the quinoid unit with a blue shift indicating the availability of Cu chromophores. However, the surface plasmon absorption (SPA) of Cu particles is 500 nm which is absent in the optical absorption of the Cu(I)-PANI composite. This could be mainly due to the presence of Cu content, which is less and has a strong absorption of PANI at a 600–660 nm range, overlapping the SPA of Cu particles.

**Figure 3:**
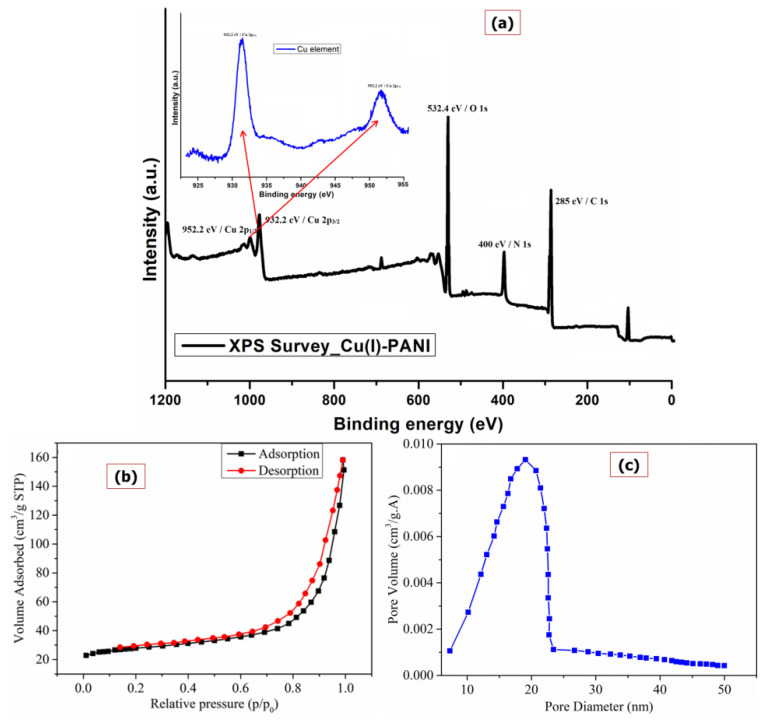
(**a**) XPS analysis, (**b**) BET surface area, and (**c**) pore size distribution curve of Cu(I)-PANI composite.

The XPS analysis for the Cu(I)-PANI composite is provided in Figure 3a where the survey spectrum confirming the presence of Cu element along with other elements of C, O, and N from the PANI polymer. In addition, the elemental Cu 2p spectrum provided in the inset is indicating for the presence of two different peaks, one at 932.2 eV for the Cu 2p3/2 and the other at 952.2 eV for the Cu 2p1/2 orbital confirming that the Cu is in its +1 oxidation state only [19,27]. Thus, from the XPS analysis, it can be confirmed for the successful formation of Cu-PANI composite with the oxidation state of Cu to be +1, but not in +2.

The BET isotherm of Cu(I)-PANI composite is shown in Figure 3b, simulating Type IV isotherm to the hysteresis loop of type H3 dependent on IUPAC classification [28]. It implies that the formed polymer composite material has a large aperture and also contains porosity. The adsorption-desorption isotherms of material are responsible for showing the type H3 hysteresis loop on it, inferring that the capillary condensation is occurring along with the mesopores. The surface area value to the Cu(I)-PANI composite is 20.5 m^2^/g, total pore volume of 0.15 cm^3^/g, and an average pore diameter of 25.34 nm was calculated. Such an observation of values refers to the fact that the Cu(I)-PANI composite has a mesoporous structure.

The BET adsorption analysis exhibited that the pore size distribution has an inverse relationship between pore diameter and surface-area density. The pore distribution of material clarifies that the surface is engrossed by a comparatively more minor mesopore structure that ranges between 7 and 20 nm (as shown in Figure 3c). The pore volume also shows a similar pattern to that of pore size since the high surface area and porosity influences the composite’s performance; and so, these are considered to be the significant parameters of solidstate materials [29].

### 3.2. RO16 Adsorption Studies

#### 3.2.1. Effect of pH

**Figure 4:**
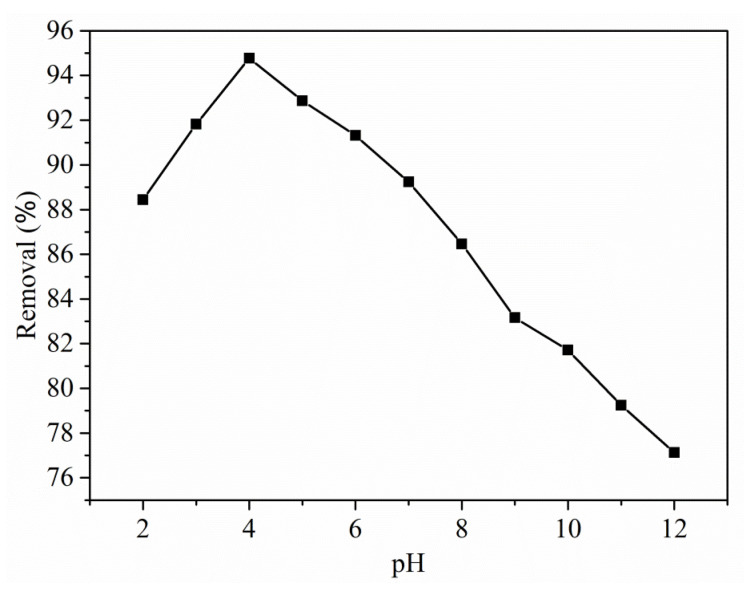
Effect of pH (2–12 range) on the removal efficiency of Cu(I)-PANI composite (the initial concentration, contact time, volume of solution, and amount of adsorbent are 100 mg/L, 90 min, 100 mL, and 0.06 g/L, respectively).

Solution pH is a significant factor for ionization dissolved in a solution, adsorbent surface, dye molecular structure, and for the separation of various functional groups on active sites [30]. Figure 4 shows the comparison of pH effect towards the removal of RO16 dye in the range of 2–12. 

From the graph, the removal efficiency of RO16 is raised by moving the pH from 2 to 4, and tends to be decreased beyond pH 4. At this pH, the maximum % removal for the RO16 dye was observed to be 94.77% and so, all the successive experiments on the adsorption process were performed at an optimal pH of 4. Further, when the pH is increased from 5 to 12, the % removal of RO16 dye is decreased. This tendency can be mainly due to the competition between extra OH^−^ ions and negatively charged dye molecules. In reality, since the pKa of RO16 is 3.75, the anionic species were the preponderant RO16 species in the solution at pH levels above this value. The most significant amount of RO16 was adsorbed at pH 4, which had a q_e_ 157.95 mg/g, followed by pH 12, which had a q_e_ 128.55 mg/g and was the lowest.

The point of zero charges (PZC) can be formed when the pH surface charge tends to zero, and such PZC is employed to define the surface’s electrokinetic features. The pH_pzc_ value is 4.92. The pH value is applied for describing the PZC of the system, where H^+^/OH^−^ is performing its potentials in determining the ions. The anionic dye adsorption process is favored at pH<pH_pzc_ because of the availability of functional group OH^−^ and the surface tends to become positively charged [31].

#### 3.2.2. Effect of Dose

**Figure 5:**
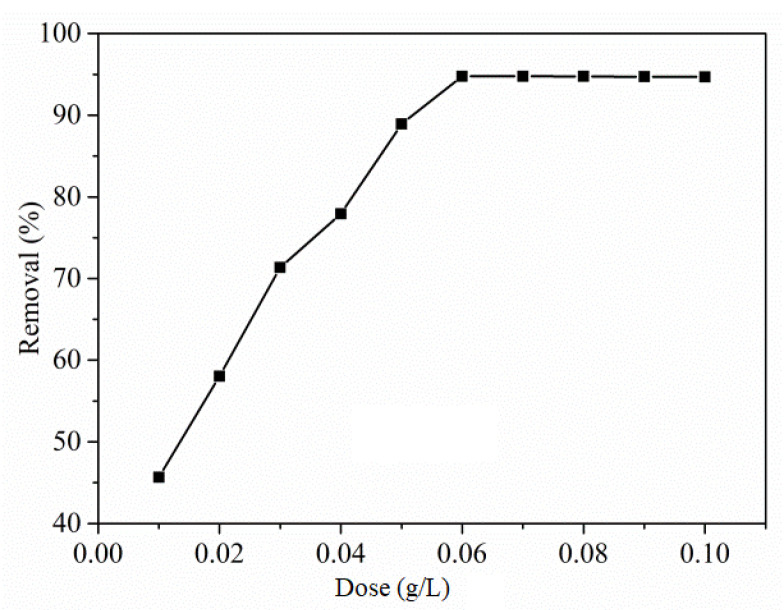
Effect of adsorbent dosage on the %removal efficiency with Cu(I)-PANI composite (initial concentration, pH, contact time, and volume of solution are 100 mg/L, 4, 90 min, and 100 mL, respectively).

The critical aspect of adsorbent dosage determines the adsorbent capacity and in that view, the Cu(I)-PANI composite dose in the range of 0.01 to 0.1 g/L was tested at a concentration of 100 mg/L dye solution to measure the % removal of RO16, as shown in Figure 5. 

From the graph, it is easy to understand an increase in the % removal of RO16 dye with that of increased adsorbent dose until 0.06 g/L, and this can be linked to the availability of a higher number of adsorbing sites in the composite. However, with further increase in the adsorbent dose beyond 0.06 g/L, no significant increase in the sorption is observed and may be the reason that the sorption amount becomes constant due to accumulation of adsorbents.

From the study, it was observed that the RO16 % removal efficacy tends to be increased at the optimum dose, and after that, there was no change in the efficiency. This could be mainly due to the fixed initial concentration of solute or an increase in the adsorbent dosage, leading to an increased surface area and linked adsorption sites [32]. When the adsorbent concentration is least, the surface becomes saturated by the dyes, where the residual dye concentration tends to become highest. Therefore, it was observed that the optimal dose for the adsorption of RO16 was 0.06 g/L, which was taken into account for carrying out further experiments.

#### 3.2.3. Effect of Initial Concentration and Contact Time

**Figure 6:**
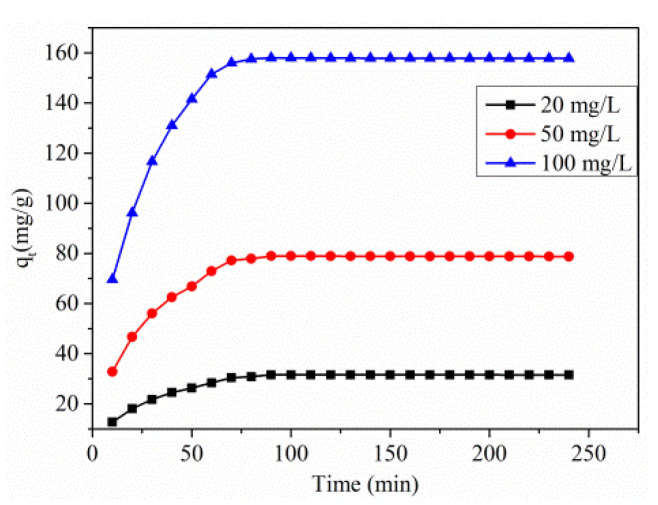
Effect of contact time on the adsorption capacity of Cu(I)-PANI composite (different initial concentration, pH, dosage, contact time, and volume of solution are 20–100 mg/L, 4, 0.06 g/L, 240 min, and 100 mL, respectively).

The contact time is considered as a significant aspect in adsorption studies, as it provides information about the kinetics of total interaction occurring at the surface of the adsorbent with that of the adsorbent. The contact time of dye adsorption for 240 min at an initial concentration of 100 mg/L and optimal pH is shown in Figure 6. 

From the figure, the time variation plots clarify that the dye removal efficiency is the starting stage, but there is a gradual slow down in the removal process when it reaches a stage of equilibrium. It is mainly because of the vacant surface sites at the preliminary stage. After some time, the vacant sites become occupied by the dye molecules, creating repulsive force in between the adsorbate and bulk phase. The equilibrium is achieved when the solution is agitated with adsorbent for 90 min. Moreover, the equilibrium showed no change following the time. Such an action means that once the equilibrium is attained, there is no chance of removing any treatment. 

In batch adsorption, the removal rate of adsorbate is controlled through the transportation of dye molecules present in the nearby sites to the internal sites of particles [33]. Hence, from the above context, the amount of absorbed dye changes with the variation of initial dye concentration, and increases with that of an increased dye concentration. As the equilibrium is attained, the amount becomes constant because the driving force is offered from a rise in the solute concentration, enabling it to overcome the resistance towards mass transfer between solid and liquid phases. Therefore, the adsorption tends to increase at higher initial concentrations. As the initial dye concentration increased from 20 to 100 mg/L at a temperature of 318 K and pH 4.0 ± 0.1, the adsorption capacity also increased from 31.55 to 157.79 mg/g. At the same time, the %removal of RO16 decreased from 94.67% to 38.2%. It is also observed that the fractional removals are high at high concentration and at low concentration, and the removal of dye is high [34].

#### 3.2.4. Adsorption Isotherm

The adsorption isotherm elaborates the relation between the amount of adsorbate taken up and the concentrated adsorbate of the remaining solution. Various equations are presented for analyzing the experimental adsorption equilibrium data. Such parameters for the equilibrium model give insight into the adsorption mechanism, features of the surface, and adsorbent affinity [35]. For the study, the Langmuir and Freundlich isotherm models are analyzed to understand which one fits well. 

**Figure 7:**
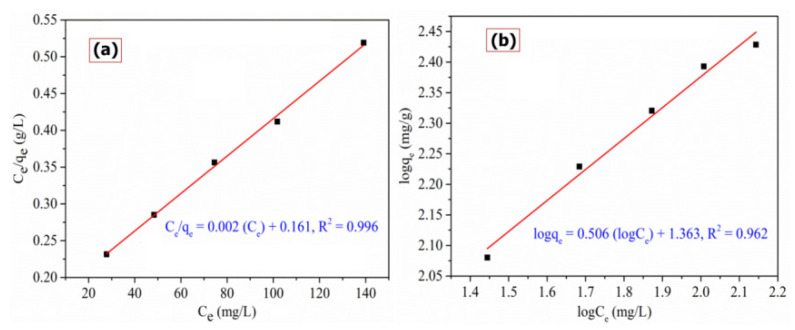
Adsorption isotherm parameters of (**a**) Langmuir, and (**b**) Freundlich model for the removal of RO16 by Cu(I)-PANI composite (initial concentration, pH, dosage, contact time, and volume of solution are 100 mg/L, 4, 0.06 g/L, 100 min, and 100 mL, respectively).

The isotherm achieved for the adsorption of RO16 dye on Cu(I)-PANI composite is shown in Figure 7a,b.

Also, the isotherm constants and correlation coefficients are estimated for the isotherm model, and the outcome of such a result is tabulated in Table 1.

From the comparative study of the linear plots of Langmuir and Freundlich models, it was observed that the *R*^2^ value of Langmuir isotherm model is 0.996, while Freundlich isotherm was 0.982 (Table 1). These *R*^2^ values indicate that the adsorption process is observed to fit well with the Langmuir isotherm model, and this also suggests that the adsorption possesses continuous adsorption energy. In other words, the Langmuir isotherm goes well with experimental data mainly because of the homogeneous distribution of active adsorption sites on the Cu(I)-PANI surface as the surface is homogeneous [36]. The maximum adsorption capacity was found to be 392.156 mg/g. The Freundlich constant, *1/n* is smaller than 1, showing that the adsorption process can be suitable to apply at optimum conditions [37].

#### 3.2.5. Kinetic Studies 

**Figure 8:**
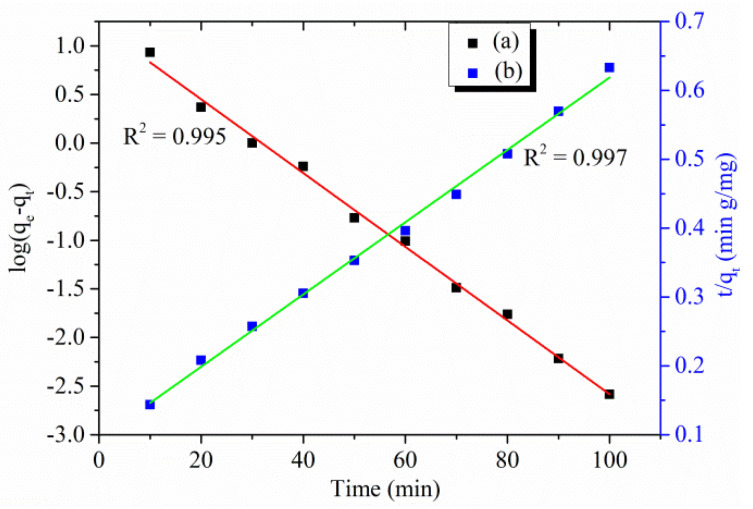
Adsorption kinetics parameter (**a**) PFO, and (**b**) PSO for RO16 adsorption by Cu(I)-PANI composite (initial concentration, pH, dosage, contact time, and volume of solution are 100 mg/L, 4, 0.06 g/L, 100 min, and 100 mL, respectively).

Adsorption kinetics is responsible for measuring the adsorption method efficiency through different applications of the kinetic model. The non-linear pseudo-first-order (PFO) is employed to determine the adsorption mechanism of dye molecules on the adsorbent surface, whereas the pseudo-second-order (PSO) can be applied to test the experimental data of initial concentration as exhibited in Figure 8. The PSO is responsible for expressing the adsorption method controlled by the chemisorption, whereas in PFO, the adsorption process is explicitly controlled by the physisorption [38].

The kinetic data were following the application of both the non-linear form of PFO and PSO and the obtained parameters are listed in Table 2. For linearized PFO, it was found that the results diverge from the straight line indicating that pore diffusion is not a controlling step for individual rate. The alteration of the *R*^2^ value from unity is a measure of incompatibility. At present, the PFO exhibits poor *R*^2^. As shown in Table 2, it was observed that the adsorption of RO16 by Cu(I)-PANI is following the PSO in a higher R^2^ value. This shows that the adsorption is a strong dependent of the presence of vacant adsorbent sites despite initial dye concentration. The PSO data make it clear that the adsorbate mechanism and adsorbent interaction coordinate with the physical attachment and the adsorption process is controlled by chemosorption.

#### 3.2.6. Thermodynamics

**Figure 9:**
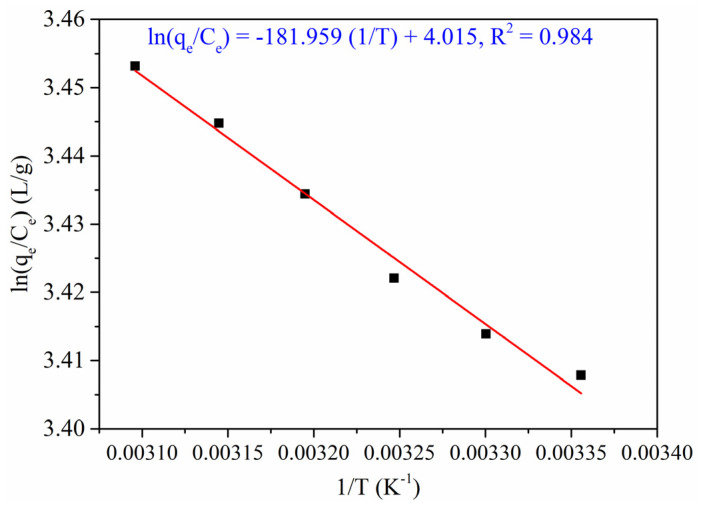
Thermodynamic study for RO16 removal by Cu(I)-PANI adsorption (initial concentration, pH, dosage, contact time, and volume of solution are 100 mg/L, 4, 0.06 g/L, 100 min, and 100 mL, respectively).

For demonstrating the feasibility of RO16 dye adsorption onto Cu(I)-PANI composite, the thermodynamic study of the adsorption process can be conducted. The values of Δ*H*° and Δ*S*° are measured from the slope and intercept of Van’t Hoff plot (lnq_e_/C_e_ vs. I/T), as shown in Figure 9 and the values calculated are tabulated in Table 3. 

From the results, the negative values of Δ*H*° confirm the fact that the dye adsorption method is exothermic and physical. There is confirmation of firm bonding on the adsorbent, which facilitates adsorbate molecules located on adsorption sites for an extended period since the molecular geometry hampers the slip–off mechanism from the adsorbent surface [39]. The negative values of Δ*G*° exhibit the feasibility and spontaneous nature of the adsorption process. In other words, the increase in a negative value of Δ*G*° decreases the temperature exhibiting the quantity adsorbed during the equilibrium, and it decreases with an increase in the temperature [40]. The value of Δ*G*° for the physisorption ranges between −20 and 0 kJ/mol, and the chemisorption process ranges from −80 to −400 kJ/mol [41]. For the corresponding study, the value of Δ*G*° is in the range of −20 to 0 kJ/mol, demonstrating that the process favors for a physisorption mechanism. 

The Δ*H*° has a positive value showing that the adsorption process is endothermic and it was also observed that the value of % removal increases with an increase of temperature from 298 to 323 K. This also confirms that the process is endothermic. The positive value of Δ*S*° established the affinity and random increase of solid solution for the adsorption of dye on the Cu(I)-PANI composite.

#### 3.2.7. Reusability

**Figure 10:**
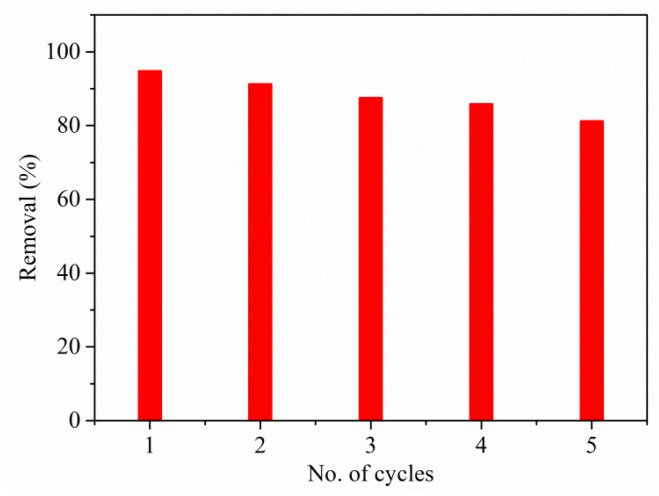
Reusability study for RO16 by Cu(I)-PANI composite (initial concentration, pH, dosage, contact time, and solution volume are 100 mg/L, 4, 0.06 g/L, 100 min, and 100 mL, respectively).

It is significant to regenerate and reuse the absorbent once it becomes saturated by dyes for practical uses. The eluent should possess different features such as activity, not be harmful to the adsorbent, non-polluting towards the atmosphere, and of course it should be cost-effective. For practical purposes, it must have two crucial factors: stability and reusability, which should be monitored thoroughly. Therefore, in the above context, the material’s reusability investigation was performed to determine the effect of material on % removal when the usage cycle is carried out repeatedly. Figure 10 shows the reusability of material for adsorption of RO16 for the five corresponding cycles. 

From the Figure 10, it is observed that there is a minute decrease in the productivity after each cycle. The % removal of RO16 for the second, third, fourth, and fifth cycles are 91.25%, 87.5%, 85.8%, and 81.2%. Such an observation of slight decrease is mainly because of the substantial weight loss that took place at the time of recovery and during purification of the adsorbent. It also leads to a decrease in the % efficiency in every repeated cycle; and, in addition, there was a reduction in active sites present during the process owing to the dye molecules that were adsorbed. The investigation found that nearly 81% of RO16 was removed in the fifth cycle, which is a significant amount, thereby indicating the higher adsorption efficiency of Cu(I)-PANI composite. The stability and reusability are important aspects of an ideal adsorbent, as they help to reduce the cost of treatment procedures, adsorbent supply, and help to minimize the adsorbent’s disposal problem. The reusability test of materials was conducted by evaluating the RO16 adsorption efficiency after treatment with four different eluents (dH_2_O, HNO_3_, (CH_3_)_2_CO, and C_2_H_5_OH). The Cu(I)-PANI stability was evaluated by measuring the concentration of Cu leachate after each adsorption/desorption cycle. From the analysis, the concentration of Cu after the fifth cycle was measured to be 0.088 mg/L, which is a negligible amount. This suggests that the Cu(I)-PANI composite has good stability towards the sequestration of RO16 dye in aqueous solutions. Finally, the analysis provides superior efficiency, stability, and reusability factors for the Cu(I)-PANI composite towards the adsorptive removal of RO16 dye that can additionally be tested for other novel dyes of long chain originics.

#### 3.2.8. Comparison with Other Adsorbents

The comparative study of the adsorption capacity of RO16 was conducted with Cu(I)-PANI composite for different adsorbents as shown in Table 4. The outcome displayed maximum adsorption capacity for the study as compared to the studies conducted with other adsorbents as reported in the literature studies [42,43,44,45,46,47,48,49,50]. Thus, the conclusion can be made that Cu(I)-PANI composite has a relatively higher adsorption capacity, indicating that it can be recognized as a suitable material to remove RO16 dye from the aqueous solutions.

## 4. Conclusions

In conclusion, we indicate the high adsorption capacity, stability, and cost-effective nature of Cu(I)-PANI composite towards the removal of RO16 dye. As per the qualitative study, it can be mentioned that the Cu(I)-PANI possesses some extraordinary ability to remove the dye from aqueous solutions as compared to the other adsorbent materials. Following the synthesis and physicochemical characterization studies, the testing of Cu(I)-PANI composite confirmed its active adsorption behavior and so serves as an alternative to the activated carbons, and also, the composite does not possess any harmful effects. With the Cu(I)-PANI composite, the highest % removal observed was 94.77% at a pH 4, maximum adsorption capacity of 392.156 mg/g, and tended to be efficient at a low cost. The Langmuir and Freundlich’s isotherms were used to analyze the equilibrium data and provided correlation for the adsorption of RO16 dye. The graph of linear equations was also used to determine the characteristic parameters for the isotherm and the correlation coefficient. The thermodynamic parameters indicated that the outcome of RO16 adsorption is spontaneous and exothermic. Based on the thermodynamic feasibilities and efficiency of removing the dyes, the formed Cu(I)-PANI can best be suitable for the adsorptive removal of RO16 dye and other adsorbents. 

## Figures and Tables

**Table 1:** Isotherm parameters for the adsorption of RO16 dye onto Cu(I)-PANI composite.

Langmuir Isotherm			Freundlich Isotherm		
q_m_ (mg/g)	K_L_ (L/mg)	*R* ^2^	n	K_F_ (mg/g)(L/mg)^1/n^	*R* ^2^
392.156	0.015	0.996	1.974	23.075	0.982

**Table 2:** Kinetic parameters for RO16 dye adsorption onto Cu(I)-PANI composite.

Pseudo-First Order			Pseudo-Second Order		
q_m_ (mg g^−1^)	k_1_ (min^−1^)	*R* ^2^	q_e_ (mg g^−1^)	k_2_ (g mg^−1^ min^−1^)	*R* ^2^
16.225	0.087	0.995	190.476	2.946 x 10^−4^	0.997

**Table 3:** Thermodynamic adsorption parameters of RO16 dye by Cu(I)-PANI at different temperatures.

Temp (K)	Δ*G* (kJ/mol)	Δ*H* (kJ/mol)	Δ*S* (J/mol K)	Removal (%)
298	−8.437	1.512	33.387	94.77
303	−8.604			94.80
308	−8.771			94.84
313	−8.938			94.90
318	−9.105			94.95
323	−9.272			94.99

**Table 4:** Comparison of maxima adsorption capacities for RO16 taken up.

S. No.	Adsorbent	Adsorption Capacity (mg/g)	Isotherm	Dye Concentartion (mg/L)	Kinetic	Ref.
1.	Chitosan cross-linked (beads)	30	Langmuir	10–70	PSO	[42]
2.	Carbonized fish scales	54.940	Freundlich	25–400	PSO	[43]
3.	Digested sludge	159	Langmuir	0–5000	PSO	[44]
4.	Paper sludge activated carbon	178	Langmuir	50–350	PSO	[45]
5.	Cross–linked chitosan/sepiolite	190.965	Langmuir	25–400	PSO	[46]
6.	Polyaniline/bacterial extracellular polysaccharides	293.2	Langmuir	-	PSO	[47]
7.	Chitosan-polyaniline	333.3	Langmuir	25–125	PSO	[48]
8.	Crosslinked chitosan-epichlorohydrine thin film	356.20	Langmuir	25–350	PSO	[49]
9.	Activated carbon from wood	367.5	Langmuir	-	PSO	[50]
10.	Cu(I)-PANI	392.156	Langmuir	100–300	PSO	Present work

## Data Availability

The data can be provided upon request from the corresponding author.
